# Risk factors of pituitary disorders following traumatic brain injury

**DOI:** 10.1186/cc12270

**Published:** 2013-03-19

**Authors:** F Lauzier, O Lachance, I Cote, B Senay, P Archambault, F Lamontagne, A Boutin, M Shemilt, L Moore, F Bernard, C Gagnon, D Cook, AF Turgeon

**Affiliations:** 1 Universite Laval, Quebec, Canada; 2 Universite de Sherbrooke, Canada; 3 Universite de Montreal, Canada; 4 McMaster University, Hamilton, Canada

## Introduction

Pituitary disorders following traumatic brain injury (TBI) are frequent, but their determinants are poorly understood. We performed a systematic review to assess the risk factors of TBI-associated pituitary disorders.

## Methods

We searched MEDLINE, Embase, Scopus, The Cochrane Library, BIOSIS, and Trip Database, and references of narrative reviews for cohort, cross-sectional and case-control studies enrolling at least five adults with TBI in whom ≥1 pituitary axis was tested and one potential predictor reported. Two independent investigators selected citations, extracted data and assessed the risk of bias. We pooled the data from all studies assessing a specific predictor, regardless of the pituitary axis being evaluated. When more than one pituitary axis was assessed, we used the data related to hypopituitarism or the data from the most defective axis. When a pituitary axis was evaluated several times, we used assessment farthest from the injury. A meta-analysis was performed using random effect models and *I*^2 ^was used to evaluate heterogeneity. Studies were considered at low risk of bias if the authors defined inclusion/exclusion criteria, did not use voluntary sampling, and tested > 90% of patients with proper detailed diagnostic criteria.

## Results

Among 13,559 citations, we included 26 studies (1,708 patients). Increased age was associated with pituitary disorders (weighted mean difference = 3.2, 95% CI = 0.3 to 6.1, 19 studies, 1,057 patients, *I*^2 ^= 59%). This finding was no longer significant when only considering studies with low risk of bias. TBI severity was associated with an increased risk of developing pituitary disorders (RR = 1.49, 1.24 to 1.77, *I*^2 ^= 17% for moderate/severe vs. mild TBI; RR = 1.78, 1.09 to 2.91, *I*^2 ^= 80% for severe vs. mild/moderate TBI), while sex was not (RR for male = 1.05, 0.98 to 1.13, 15 studies, 870 patients, *I*^2 ^= 0%). Among CT scan findings, only skull fractures were associated with pituitary disorders (RR = 1.75, 1.13 to 2.70, six studies, 345 patients, *I*^2 ^= 47%). An insufficient number of studies with low risk of bias assessing the association between GCS, CT scan findings and pituitary disorders was retrieved to perform meta-analysis.

## Conclusion

Age, TBI severity and skull fractures are associated with an increased risk of pituitary disorders. Further studies are necessary to identify additional factors that will help developing targeted screening strategies.

